# Fully Bio-Based Hybrid Composites Made of Wood, Fungal Mycelium and Cellulose Nanofibrils

**DOI:** 10.1038/s41598-019-40442-8

**Published:** 2019-03-06

**Authors:** Wenjing Sun, Mehdi Tajvidi, Christopher G. Hunt, Gavin McIntyre, Douglas J. Gardner

**Affiliations:** 1 School of Forest Resources and Advanced Structures and Composites Center, University of Maine, Orono, ME 04469 USA; 2 USDA Forest Products Laboratory, 1 Gifford Pinchot Drive, Madison, WI 53726 USA; 3 Ecovative Design, LLC., 70 Cohoes Avenue, Green Island, NY 12183 USA

## Abstract

Novel hybrid panel composites based on wood, fungal mycelium, and cellulose nanofibrils (CNF) were developed and investigated in the present study. In one set of experiments, mycelium was grown on softwood particles to produce mycelium-modified wood which was then hybridized with various levels of CNF as binder. The other set of experiments were conducted on unmodified wood particles mixed with CNF and pure mycelium tissue. It was found that the composites made of mycelium-modified wood and CNF resulted in enhanced physical and mechanical properties compared to the ones made by physically mixing wood, mycelium, and CNF. Scanning electron microscopy (SEM) images showed that mycelium modification covered wood particles with a network of fungal hyphae whereas CNF formed a uniform mycelial film over wood particles. Mycelium modification had a significant effect on reducing water absorption and thickness swelling of the hybrid composites and CNF increased the modulus of rupture and modulus of elasticity, optimally at 2.5% addition. We also present results and analysis pertaining to the development of unique lightweight composite systems with physical and mechanical properties optimized at 5% CNF addition with potential to be used in packaging and furniture applications.

## Introduction

Lignocellulosic-based composites are receiving greater attention in recent years because they are renewable, biodegradable, and often eco-friendly compared with synthetic materials. However, the most commonly used adhesives used to bind those natural particles are formaldehyde-based resins^1^ which limit the development of 100% natural-based composites. Moreover, formaldehyde emissions have been categorized as carcinogenic and toxic to humans^2,3^. Limiting formaldehyde emissions from wood composite products is preferred in most applications^4–8^. Recently, the US Environmental Protection Agency (EPA) finalized a rule to reduce exposure to formaldehyde emission from certain wood materials produced domestically or imported into the United States. These products include hardwood plywood, medium-density fiberboard, particleboard as well as household and other finished goods containing these products^9^.

The alternatives of formaldehyde-bonded composites are composites bonded by formaldehyde- free synthetic resins, natural-based resins and self-binding composites^10–15^. Common natural-based resins that have been developed include carbohydrates, proteins, lignin, tannins, and synthetic molecules from natural sources rather than petroleum^16–21^. Most recently, cellulose nanofibrils (CNF) have been demonstrated as a binder in conventional and novel composite systems. CNF has extremely high surface area and can bond wood particles and fibers through hydrogen bonding and mechanical interlocking, providing structural integrity to the composites^22–26^.

The technologies of producing self-binding composites include chemical or enzymatic pretreatments^27,28^, steam explosion^29^, steam injection pressing^30^ and others. Enzymatic pretreatments basically treat wood or other agricultural fiber residues with phenol-oxidizing enzymes (laccase, peroxidases etc.) derived from white rot fungi or other sources^31^. It is proposed that lignin is depolymerized during treatment and re-polymerizes during hot pressing. This technology has been used in different industries including textile, paper, wood, food and organic synthesis^10^.

Recently, mycelium-based biopolymer composites have been commercialized^32^. Mycelia of filamentous fungi digest and bond to the surface of lignocellulosic materials, form entangled networks and provide mechanical strength to panels with fire resistance and acoustical absorption properties. So far, most of the studies have been focused on low-density, foam-like unprocessed mycelium-based composites^32–36^ however Pelleter *et al*.^37^ densified low-density (0.42 g.cm^−3^) mycelium-based composites using a heat treatment process. The densified composites achieved five levels of density and also had acoustic absorption properties. If this unique technology can be applied to traditional wood-based composites (fiberboard, particleboard etc.) manufacturing, there will be additional applications and markets for this interesting natural material.

**Figure 1:**
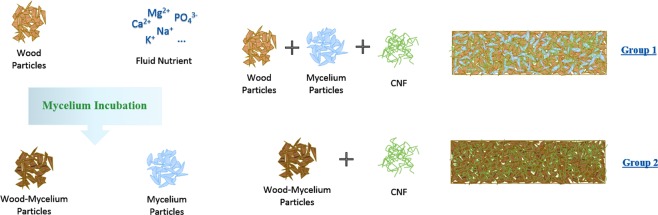
Schematic representation of the materials and composite production.

The present work introduces a panel system that incorporates wood particles treated with fungus where additional bonding is provided by CNF. We aimed to investigate the physical properties of a 100% bio-based particleboard-like hybrid composite by combining the binding capacity of CNF and mycelium together. The adhesion mechanism of mycelium bonding was also explored using two material systems as shown in Fig. 1. In Group 1, pure mycelium was grown in nutrient substrate and was mixed with pure wood particles after thermal inactivation. The mixture was used as basic material for hybrid composite manufacturing. In Group 2, the mycelium was directly grown in wood particles and the partially decayed wood particles were inactivated as well and then used as basic material for the other set of experiments. The production procedures were forming, cold-pressing, and hot-pressing. The morphology of the materials, the physical and mechanical properties of composites with different material combinations, and densities were also investigated.

## Experimental Section

### Materials

The wood particles were a commercially available mixture of spruce, pine, and fir (SPF) particleboard particles, provided by Ecovative Design LLC (Green Island, NY, USA). The pure white-rot basidiomycete mycelium tissue was grown in a solid state fermentation process at the Ecovative Design facility in Green Island, NY, USA. The tissue was grown aerially in a proprietary incubation environment such that it grew up and out of the substrate with a loft height of 75 mm. The tissue was then harvested and dried at 43 °C to deactivate the fungus. The wood-mycelium particles (WM) were produced by growing the same fungus on SPF softwood particles using the same procedure as growing the pure mycelium.

Cellulose nanofibrils (CNF) were the product of the University of Maine’s Process Development Center (PDC). Characteristics of the CNF used in this study are provided elsewhere^38–40^. Briefly speaking, bleached softwood kraft pulp fibers were circulated through a refiner until the fines content was over 90% as determined by laser diffraction as fibers smaller than 200 microns. The original concentration of CNF was 3% wt.

### Hybrid composite manufacturing

To investigate the bonding mechanism of the wood-mycelium particle systems, two different forms of wood and mycelium mixture were used as a basic combination for hybrid composite manufacturing. The first is directly mixing wood particles and pure mycelium; the second is using wood-mycelium particles produced from growing mycelium on wood particles. These two basic mixtures were combined with CNF in different percentages as shown in Table 1. The particle sizes of the wood materials used in both groups were 1.40–3.50 mm screened through a vibrating sieve. The target density was 0.6 g.cm^−3^, with thickness of 9.4 mm, controlled by stops in the hot press.

**Table 1:** The experimental design of hybrid composite manufacture (Group 1: wood particles + mycelium + CNF; Group 2: wood-mycelium particles + CNF).

Group	Label	Materials		
		Wood Particles (%)	Mycelium (%)	CNF (%)
Group 1	90W10 M	90	10	0
	90W7.5 M	90	7.5	2.5
	90W5 M	90	5	5
	90W2.5 M	90	2.5	7.5
	90W0 M	90	0	10
		**Wood-Mycelium Particles (%)**		**CNF (%)**
Group 2	100WM	100		0
	97.5WM	97.5		2.5
	95WM	95		5
	92.5WM	92.5		7.5
	90WM	0		10

To further investigate the utilization of wood-mycelium particles and CNFs system in lightweight structures, additional composites with different densities and CNF contents were manufactured. Details are shown in Table 2. For all groups, five replicate panels were manufactured.

**Table 2:** The experimental design of lower-density hybrid composite manufacture.

Group	Label	Wood-Mycelium Particles (%)	CNF (%)	Density (g cm^−3^)
Effect of Density Comparison	0.3	97.5	2.5	0.3
	0.4	97.5	2.5	0.4
	0.5	97.5	2.5	0.5
	0.6	97.5	2.5	0.6
Low-density Optimization	0.4–97.5WM	97.5	2.5	0.4
	0.4–95WM	95	5	0.4
	0.4–92.5WM	92.5	7.5	0.4
	0.4–90WM	90	10	0.4

Different components of the raw materials were mixed using a stand mixer with a paddle mixing blade, at speed 2 (KitchenAid, St. Joseph, MI, USA) for 2 mins. To make comparisons with the control formulation where CNFs were not used, equivalent amounts of water were added to the mixture. This was done to eliminate the effect of water on the properties of produced panels as CNF was a suspension in water and could not be used in dry form. Then the mixture was evenly distributed into a 120-mm square aluminum forming box. The formed mixture was first cold pressed using a hydraulic press (Dake Corporation, Grand Haven, MI, USA) to remove approximately 50% of the water. The cold press pressure was around 400 kPa and the solid contents of the mats before and after cold press were approximately 16% and 38%, respectively. The dewatered mat was then hot pressed (Carver, INC., Wabash, IN, USA) at 180 °C for 15 min to produce final hybrid panels.

### Composite Panel Characterization

#### Material morphology

The nanostructure of CNF was viewed by transmission electron microscopy (TEM) (CM10 TEM, Philips, Amsterdam, Netherlands). Drops of 0.001 wt. % CNF suspensions were deposited on carbon-coated electron microscopy grids and negatively stained with 1% uranium acetate. The grids were dried in air and observed at an acceleration voltage of 80 kV. The morphology of the wood particles, wood-mycelium particles, pure mycelium and different combinations of the mixture were studied by a scanning electron microscope (SEM) (Amray 1820, Amray Inc., New Bedford, MA, USA) with an acceleration voltage of 10 kV. The samples were placed on specimen mounts with double-sided carbon tape and grounded on all edges with conductive silver paint and sputter coated with 23 nm of gold-palladium.

#### Thermal stability analysis

The thermal stability evaluation of the raw materials was carried out under nitrogen gas on a TA Instruments TGA Q500 (TA Instruments, New Castle, DE, USA) with a high resolution (Hi-Res) option from room temperature to 600 °C. In the Hi-Res approach, the heating rate is dynamically and continuously modified, ranging from 0.001 °C min^−1^ to the maximum heating rate (20 °C min^−1^) in response to changes in the decomposition rate of the sample. The Hi-Res option is used to differentiate overlapping or close decomposition peaks. The resolution and sensitivity settings were 4.0 and 1.0 °C, respectively. The TGA results are shown as the variation of the sample mass (TG) or as a derivative weight loss (DTG) curve corresponding to the temperature.

#### Particle size distribution and dimensional analysis

The wood and wood-mycelium particles were well dispersed on a sheet of paper and the images were scanned by a Canon Document Feeder (DADF-AP1, Canon, Inc., Tokyo, Japan) with a resolution of 600 dpi. The original color images were first converted to black background using Photoshop software (Photoshop CC, Adobe Systems, Mountain View, CA, USA) and binary image using Image J software^41^ (ImageJ 1.48 v, National Institutes of Health, USA). The basic geometrical attributes of the particles including length, width, area and perimeter were analyzed by ImageJ based on the best-fitting ellipse. Three shape factors were also calculated as aspect ratio, circularity and roundness^42^. A minimum of 500 particles of each sample were analyzed.

#### Water absorption and thickness swelling

The water absorption and thickness swelling of different composites were measured according to ASTM D1037 (2012)^43^ with modifications using 3 × 3 cm specimens (8 replicates were used in each group). The specimens were immersed in distilled water and the weights and thicknesses were measured after 2 and 24 h. The water absorption and thickness swelling values were determined from the weight and thickness difference in relation to initial weight and thickness.

#### Mechanical testing

The modulus of rupture, the modulus of elasticity and the internal bond strength were determined according to ASTM D1037 (2012)^43^ with modifications using an Instron 5966 universal testing machine (Instron, Norwood, MA, USA) with a 10 kN load cell capacity. For the three-point bending test, rectangular specimens measuring 12 × 3 cm were tested using a span of 80 mm and a cross-head speed of 3 mm min^−1^. For the internal bond strength tests, the dimensions of the specimens was 3 × 3 cm and the cross-head speed was 0.4 mm min^−1^. Eight replicates were tested in each group.

### Statistical analysis

The obtained data were analyzed using IBM SPSS Statistics Version 23 (IBM Corp., Armonk, NY, USA). Because the variables were different in the two groups of experiments, one-way analysis of variance (ANOVA) was used to determine the differences between the group means for the two groups separately. A Duncan’s Multiple Range Test (MRT) test was then performed to further assess the significance level of the mean values for each treatment level. All comparisons were made at 95% confidence level.

## Results and Discussion

### Characterizations of raw materials

**Figure 2:**
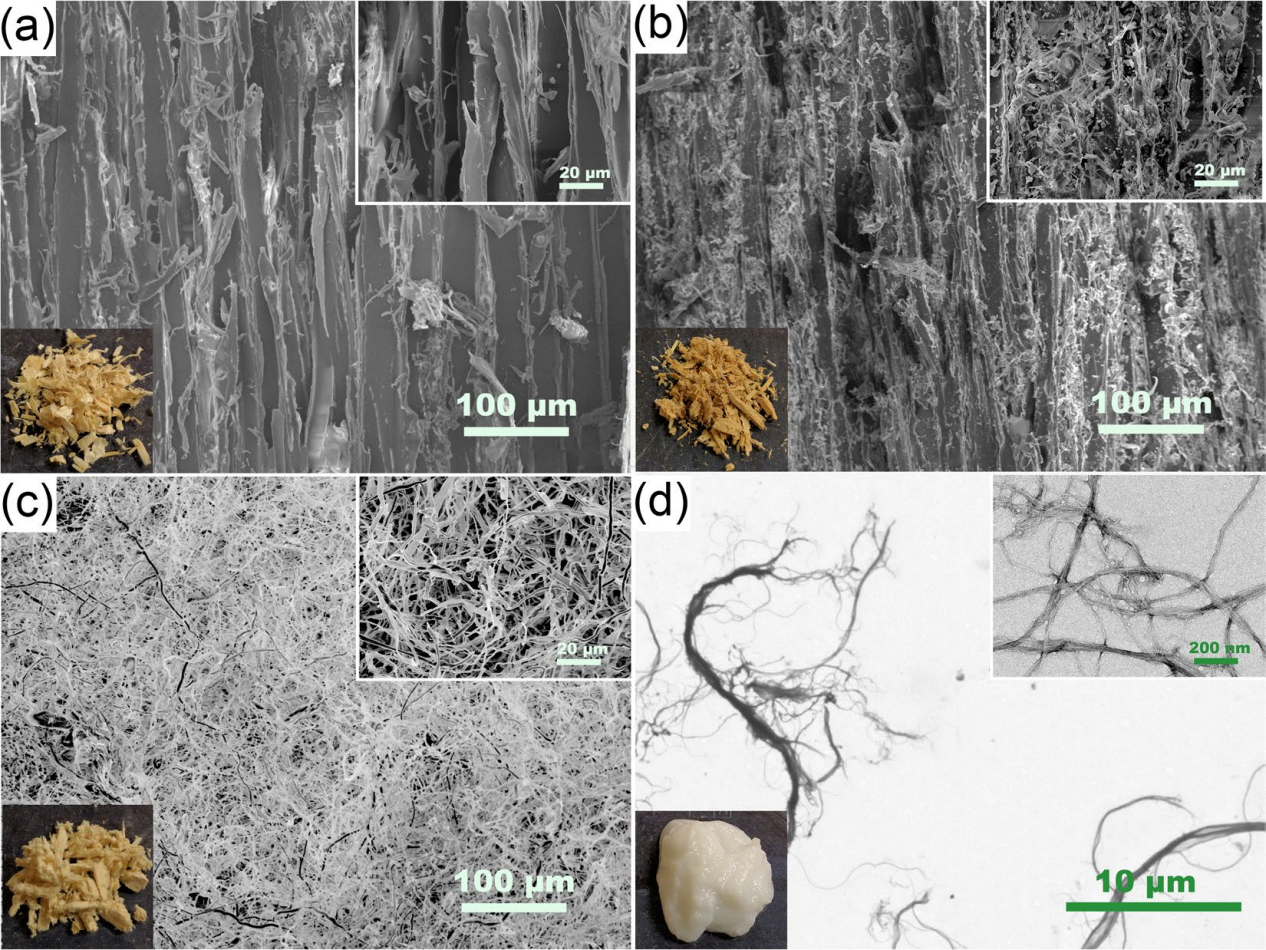
Morphology of raw materials: wood particles (**a**), wood-mycelium particles (**b**), pure mycelium (**c**), and CNF (**d**).

The morphology images of raw materials are shown in Fig. 2. The surfaces of the original tracheids in the wood particles are very smooth (Fig. 2(a)). After mycelium colonization, smooth cell walls are covered by a fibrous network of fungal hypha and the color of the wood particles turned darker (Fig. 2(b)). At the initial stage of wood decay, the fungi hyphae penetrate wood, initiate colonization, and release enzymes^44^. The brownness is a common change attributed to the synthesis of melanin at the early stage of wood decay^44,45^. As shown in Fig. 2(c), the aerial mycelium grown in a solid state fermentation method has a porous structure composed of tubular hyphae. This structure was not apparent on the wood-mycelium particle surfaces (Fig. 2(b)). Under TEM, CNF appeared as thin elongated and branched fibers with multiple ramifications and sub-ramifications, which would easily form networks upon drying (Fig. 2(d))^46,47^.

**Figure 3:**
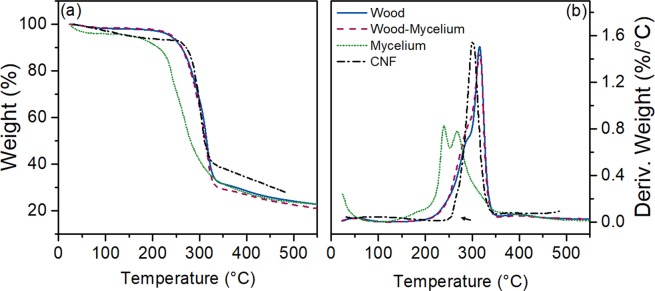
TG (**a**) and DTG (**b**) curves of composite raw materials.

The thermal degradation profiles of raw materials reveal that most of the degradation occurred between 200 and 350 °C (Fig. 3). Pure mycelium showed the lowest thermal stability and started to degrade at around 208 °C (T_on_). The two peaks appearing at 239 °C (T_P1_) and 269 °C (T_P2_) (Table 3) in the DTG curve match the reported degradation of carbohydrates and proteins, respectively, in the mycelium^48^. Both the TG and DTG curves of wood-mycelium particles appear similar to the curves of wood particles, showing that the weight percentage of mycelium in wood particles was very low. Compositional TGA analysis performed on mixtures of wood particles and mycelium (data not presented here) showed that the weight percentage of mycelium in the in wood particles was less than 10%. The degradation by fungi had very little influence on the thermal stability of wood particles. Compared with wood particles, which started to degrade at around 244 °C (T_on_, or initial rise in 3b), with a maximal degradation temperature of 316 °C (T_P2_), the thermal degradation onset of CNF occurred at a higher temperature at 257 °C (T_on_), with one degradation peak at 301 °C (T_P2_). This increase in thermal stability (higher onset temperature in Fig. 3b, indicated by arrow) is proposed to be caused by the removal of hemicellulose, lignin, pectin and other less stable components in wood^49^.

**Table 3:** Onset and peak temperatures of thermal degradation for wood particles, wood-mycelium particles, pure mycelium and CNF obtained from DTG data (Fig. 3b).

Materials	^a^T_on_ (°C)	^b^T_P1_ (°C)	^b^T_P2_ (°C)
Wood	243.8	286.2	316.0
Wood-Mycelium	241.9	283.0	315.1
Mycelium	207.9	239.0	268.9
CNF	257.2	—	301.0

^a^The onset temperature T_on_ was estimated by the intersection of the tangent lines in 4b.

^b^The degradation temperature T_P1_ and T_P2_ refer to the different peak temperatures observed on the DTG curves and are related to the different thermal degradation steps for each material.

**Figure 4:**
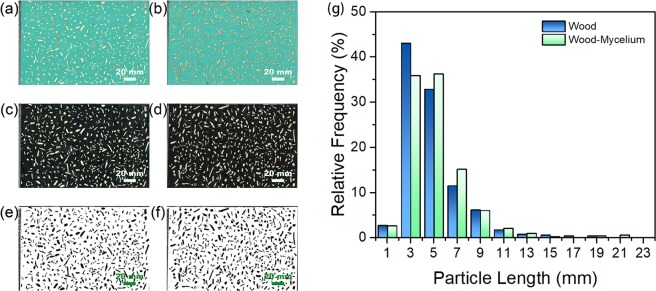
Original scanned (**a**,**b**), black background (**c**,**d**), and binary (**e**,**f**) images of wood (**a**,**c**,**e**), wood-mycelium particles (**b**,**d**,**f**) (left) and relative length frequencies of wood and wood-mycelium particles (**g**) (right).

All the wood and wood-mycelium particles used in this study were obtained after sieving. The mesh size was between 1.40 and 3.35 mm. However, particles normally pass through the sieves only based on their width^42^. By image analysis, more information about the particles can be obtained and compared. Figure 4 and Table 4 show the relative length frequencies and other dimension and shape factors of wood and wood-mycelium particles, respectively. No significant difference was found in particle length distribution between wood and wood-mycelium particles, as expected (p-value > 0.05), meaning that changes seen in physical and mechanical properties of panels were not affected by wood particle size.

**Table 4:** Dimensions and shape factors (width, length, aspect ratio, circularity and roundness) of wood and wood-mycelium particles (mean ± one standard deviation).

Parameters	Wood Particles	Wood-Mycelium Particles
Width (mm)	1.87 ± 0.77	1.97 ± 0.81
Length (mm)	4.79 ± 2.48	5.04 ± 2.65
Aspect Ratio	3.16 ± 5.62	3.11 ± 3.11
Circularity	0.46 ± 0.17	0.48 ± 0.17
Roundness	0.46 ± 0.22	0.46 ± 0.23

### Comparison of the two wood-mycelium-CNF hybrid systems

**Figure 5:**
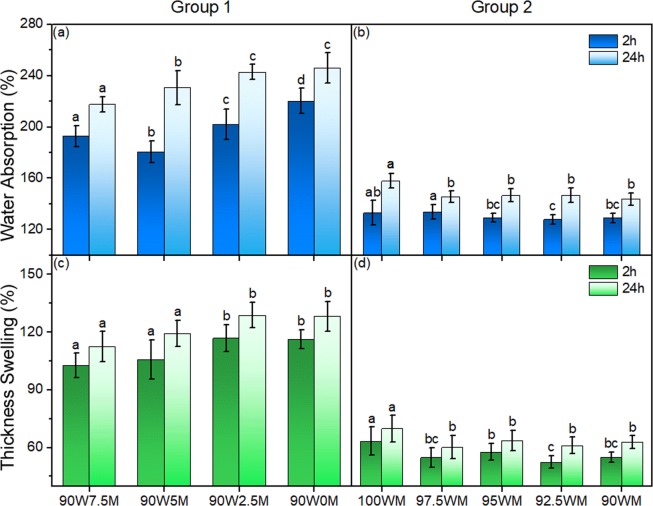
Water absorption (**a**,**b**) and thickness swelling (**c**,**d**) of Group 1 (**a**,**c**) and Group 2 (**b**,**d**). In each figure, columns with common letters are not significantly different at 95% confidence level (p-value > 0.05).

Water absorption and thickness swelling are not limiting factors for indoor type composites such as particleboard. However, more dimensionally stable products are often preferred and these two properties can also shed some light on the quality of adhesion in the panel. Figure 5 shows the water absorption and thickness swelling results of the two groups. Both water absorption and thickness swelling values are very high in Group 1 (Fig. 5(a,c)), revealing that there was not sufficient adhesion in the system. No data could be collected from the specimens with no CNF addition (90W10M) in Group 1 as the panel quickly fell apart after being immersed in water, which shows that the interactions between pure mycelium blended with wood particles are not water resistant. Conversely, specimens in Group 2 show much lower water absorption and thickness swelling values (Fig. 5(b,d)). The specimen 100WM with no CNF addition shows 158% water absorption and 70% thickness swelling after 24 h, which is significantly lower than all the specimens in Group 1. At the initial stage of wood decay, fungal hyphae grow on wood cell walls and cover the particle surfaces (Fig. 2(a,b)), which increases the surface interactions during the hot-pressing process. Enzymes typically produced by white-rot fungi can degrade lignin and produce radicals which might help improve the adhesive bonding^50–52^.

The many available hydroxyl groups on CNF form hydrogen bonds with wood, mycelium, and other CNF particles, which is one of the main adhesion mechanisms contributing to CNF bonded panels. The higher water absorption and thickness swelling of Group 1 samples with higher levels of CNF (Fig. 5a,c) is attributed to the loss of hydrogen bonds between the wood/mycelium particles during water soaking. Group 2, however, shows a small positive (reducing) effect of CNF addition on water absorption and thickness swelling (Fig. 5b,d). Common letters on the columns in the figure indicate that the means of the parameters were not significantly different at 95% confidence level; therefore the effect of adding CNF to the Group 2 samples on water absorption and thickness swelling was statistically significant (p-value < 0.05) only when 2.5% CNF was added. Further addition of CNF did not change these parameters significantly. A possible explanation is that the hydroxyl groups reacted with crosslinkers or radicals formed during fungi-induced degradation and were fixed in the entire system^31,53^.

**Figure 6:**
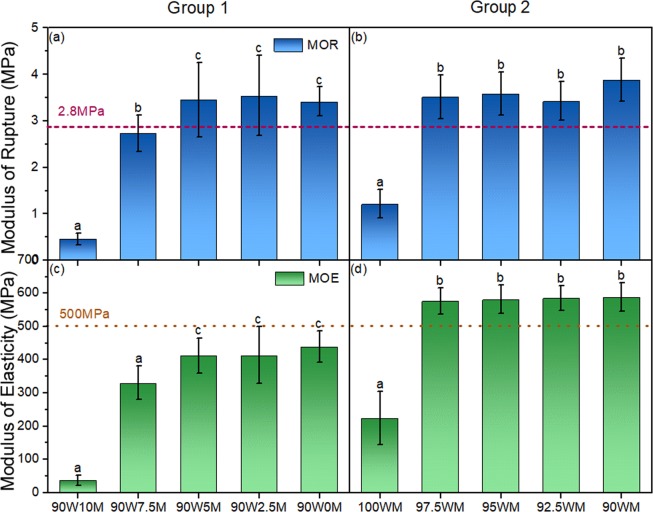
The modulus of rupture (**a**,**b**) and the modulus of elasticity (**c**,**d**) of Group 1 (**a**,**c**) and Group 2 (**b**,**d**). The horizontal lines indicate the minimum value of the modulus of rupture and the modulus of elasticity required to meet the ANSI A208.1 standard for LD-1grade^6^. In each figure, columns with common letters are not significantly different at 95% confidence level (p-value > 0.05).

Figure 6 shows the modulus of rupture and the modulus of elasticity results of Group 1 (a) and Group 2 (b). The modulus of rupture measures the ultimate load-carrying capacity while the modulus of elasticity measures the resistance to bending and reveals the stiffness of the sample^54^. These two parameters are widely used to evaluate the mechanical performance of panel products. With no CNF addition, both the modulus of rupture and the modulus of elasticity of Group 1 (90W10M) and Group 2 (100WM) are very low. Compared with 90W10M, 100WM shows higher modulus of rupture and the modulus of elasticity values indicating that treating wood particles with the mycelium positively affects CNF performance as binder. This could be because of wood composition changes caused by fungal degradation had positive effects on bonding and properties by providing more hydrogen bonding sites as corroborated by thickness swelling data presented earlier. Water can easily abrupt hydrogen boding and the fact that thickness swelling data are in agreement with mechanical performance indicates the importance of hydrogen bonding in adhesion.

The addition of CNF increased both the modulus of rupture and the modulus of elasticity in both Group 1 and Group 2 significantly (p-value < 0.05). However, there was no significant difference in the specimens of a series when the amount of CNF was higher than 5% in Group 1 and 2.5% in Group 2. This shows there may be an ultimate loading level of CNF on the surface of wood and mycelium enough for promoting adhesion beyond which no further improvement is observed^22^.

**Figure 7:**
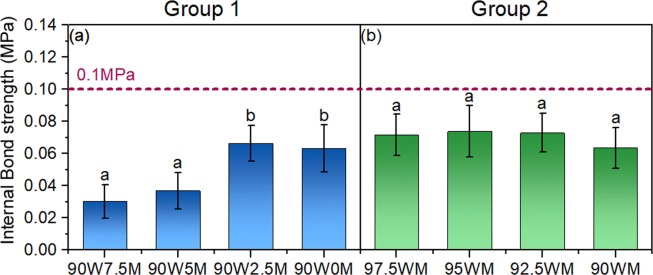
The internal bond strength of Group 1 (**a**) and Group 2 (**b**). The horizontal line indicates the minimum value of the internal bond strength required to meet the ANSI A208.1 standard for LD-1 grade^6^. In each figure, columns with common letters are not significantly different at 95% confidence level (p-value > 0.05).

Adhesion performance in wood-based panels is also quantified using the internal bond strength. The results of the internal bond strength tests are shown in Fig. 7. With no CNF addition, the composites from both groups (90W10M and 100WM) were too weak to be measured in the internal bond strength test. The internal bond strength values reached 0.03 MPa and 0.06 MPa after adding 2.5% and 7.5% of CNF in Group 1, respectively. There was no significant difference between any of the CNF levels in Group 2, which was the same as the observation in the bending test.

The horizontal lines in both Fig. 6 and Fig. 7 indicate the required values for the particleboard grade LD-1 from the US particleboard performance standard, ANSI A208.1^6^. Composites made by wood-mycelium particles and CNF in Group 2 met the standard in both the modulus of rupture and the modulus of elasticity, but the values of the internal bond strength were lower than the standard value (0.1 MPa). Previous work showed that with 15% of CNF addition to southern pine wood particles, the internal bond strength value of the composite was around 0.4 MPa^25^. The much lower the internal bond strength value in this study might be caused by the larger particle size of the raw material and less CNF used. Water absorption and thickness swelling are not included in ANSI A208.1 as particleboards are interior products not meant to be exposed to water therefore direct comparison with the standard is not possible. However, the significant reduction in these two parameters in Group 2 samples is very promising.

**Figure 8:**
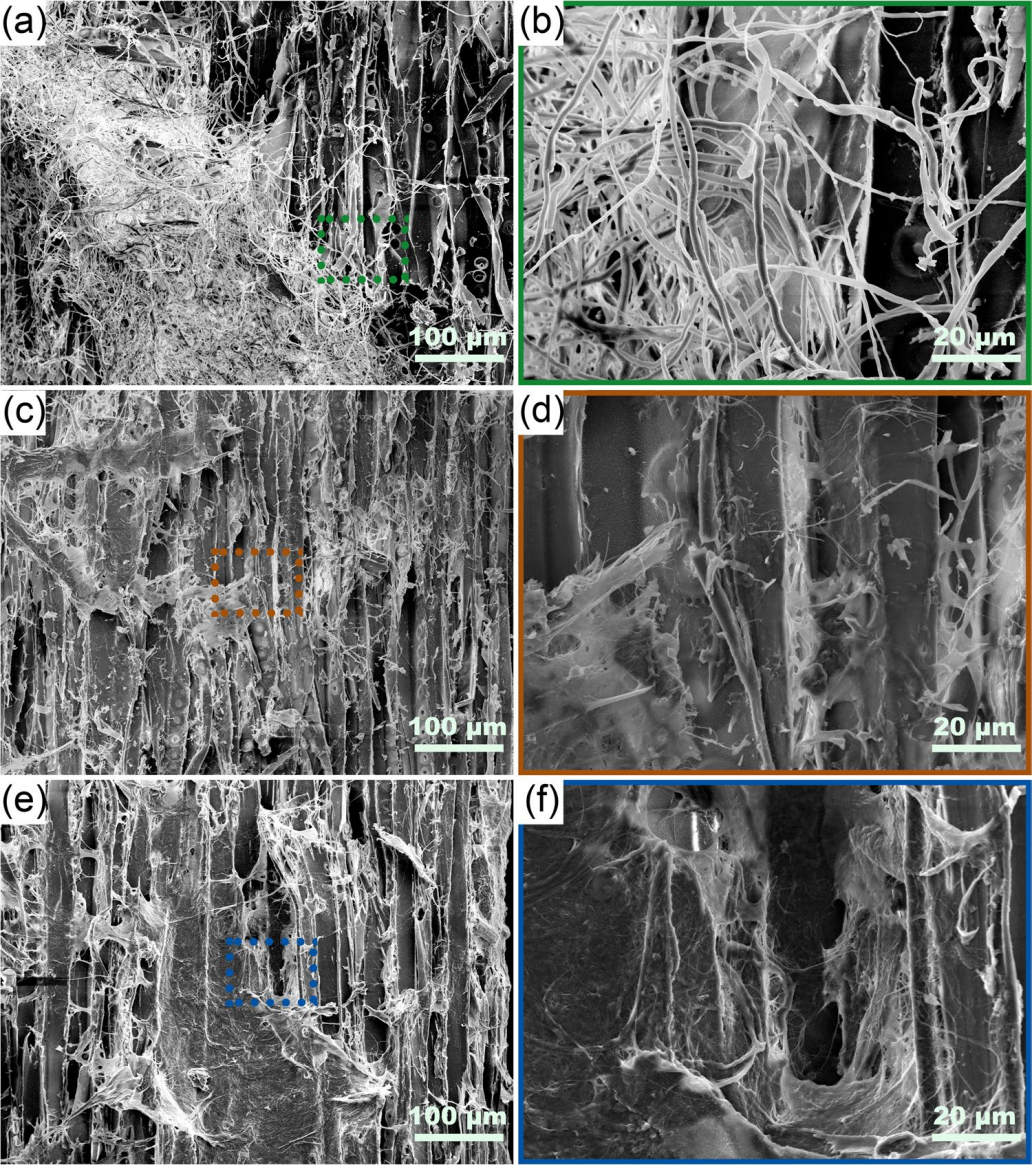
SEM images of different mixtures of raw materials with different magnifications, 200 × (**a**,**c**,**e**) and 1000 × (**b**,**d**,**f**). (**a**,**b**) 90% Wood + 10% Mycelium; (**c**,**d)** 90% Wood-Mycelium + 2.5% CNF; (**e**,**f**) 90% Wood + 10% CNF.

To further investigate the interactions among raw materials in different mixtures and explain the property difference between Group 1 and Group 2, various combinations of raw materials were mixed and dried and then observed by SEM (Fig. 8). Simply mixing pure mycelium and wood particles together did not adequately distribute the mycelium particles (Fig. 8(a,b)). Some areas of the surfaces of wood particles were covered with mycelial hyphae, similar to Fig. 2(c), while other parts remained the same as untreated wood particles seen in Fig. 2(a). This uneven distribution of mycelium is likely to be at least partly responsible for the lower properties of the composites in Group 1. Figure 8(c,d) show the morphology of wood-mycelium particles after mixing with 2.5% CNF- where the original surfaces of the particles (Fig. 2(b)) were well covered by a layer of CNF film and look much smoother. This structure looks similar to the structure of mixing pure wood particles with CNF (Fig. 8(e,f)). The fungal mycelium-covered surfaces of wood-mycelium particles have a positive effect on the deposition of CNF and the strong CNF-CNF interaction helped improve the modulus of rupture and the modulus of elasticity in both groups.

There are many possible mechanisms to explain the performance of these two hybrid systems. The better properties achieved by Group 2 than Group 1 might be caused by, but may not be limited to the following reasons^55^: (1) the better dispersion of mycelium thereby providing better adhesion, (2) the chemical changes of wood particles such as degradation of hemicellulose and lignin which may open more pores on the surface of wood cell wall, increase the surface energy and provide more functional groups for bonding and (3) the chemical differences in the structure of mycelium grown on different substrates. The SEM images clearly confirm that hypothesis 1 is reasonable but other possibilities might also be involved and are currently being studied by our research group. The CNF impressively improved the (dry) the modulus of rupture and the modulus of elasticity at low addition rates, which we attribute to its very good hydrogen bonding^55^, but had little effect on wet properties of water absorption and thickness swelling.

### Utilization of the hybrid system in lightweight composites

The experimental results in the former sections confirm that the wood-mycelium particles and CNF system (Group 2) is a viable way to achieve fully bio-based particleboard-like composites. Considering the special attention to lightweight composites in packaging, handling and transportation in recent years^56,57^, the utilization of this hybrid system in lightweight composites was further investigated. The ultimate goal was to produce lightweight composites with acceptable physical and mechanical properties using mycelium-treated wood particles.

**Figure 9:**
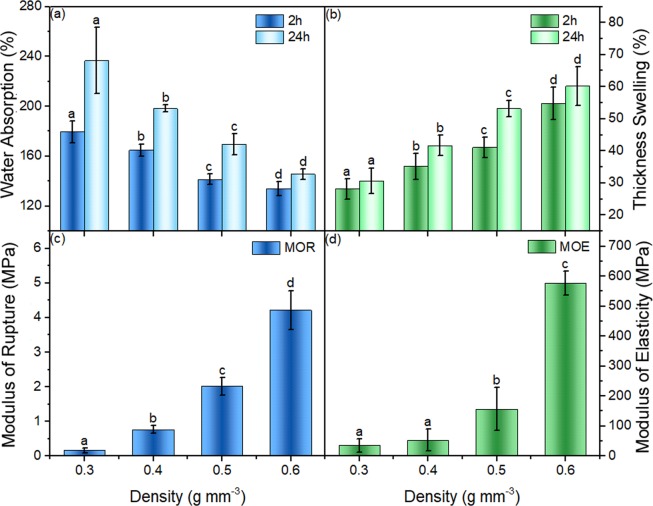
Water absorption (**a**), thickness swelling (**b**), modulus of rupture (**c**) and modulus of elasticity (**d**) of samples labeled “Effect of Density Comparison” group in Table 2. In each figure, columns with common letters are not significantly different at 95% confidence level (p-value > 0.05).

The first step was to vary densities at the same CNF addition ration (2.5 wt.%). The water absorption, the thickness swelling, the modulus of rupture and the modulus of elasticity results of the hybrid composite with 2.5 wt.% CNF addition at different densities (Table 2) are shown in Fig. 9. The 2.5% addition of CNF bonded the composites with different densities very well. The water absorption decreased with increasing composite density as a result of the decreasing the amount of voids and pores, whereas the thickness swelling, the modulus of rupture and the modulus of elasticity increased with density, as is typical^25,26^.

**Figure 10:**
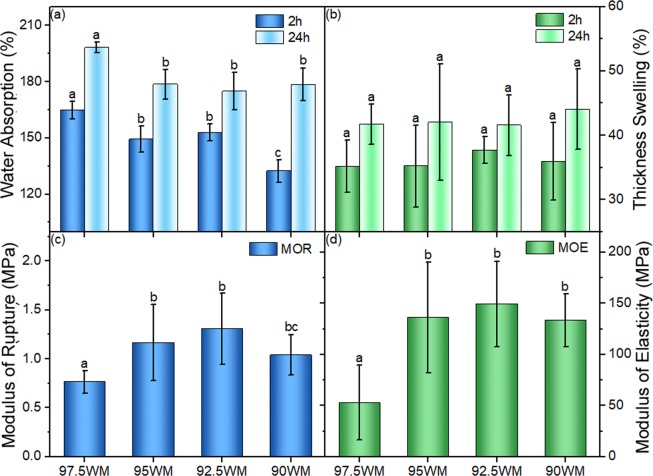
Water absorption (**a**), thickness swelling (**b**), modulus of rupture (**c**) and modulus of elasticity (**d**) of “Low-density Optimization” group in Table 2. In each figure, columns with common letters are not significantly different at 95% confidence level (p-value > 0.05).

As 2.5% CNF was found to be the optimal level for the 0.6 g.cm^−3^ density composite system, it was interesting to see if it was the same in lower density systems. Therefore, the 0.4 g1cm^−3^ density system was chosen and the hybrid composites with 2.5%, 5%, 7.5%, 10% addition of CNF were manufactured (Table 2) to optimize physical and mechanical properties. As shown in Fig. 10, the water absorption, the modulus of rupture and the modulus of elasticity were significantly higher (p-value < 0.05) at 5% CNF loading than 2.5% in these panels at 0.4 g.cm^−3^. With the decrease of density, the structure benefited from additional CNF available to enhance bonding.

Overall, hybridization of CNF and mycelium as two fully bio-based adhesive systems proved very promising. Efficient dewatering and reduction of press cycle remain important issues key to the successful implementation of CNF as binder in wet-formed composite panels. Studies on the dewatering mechanism through contact dewatering are currently underway in our research group and preliminary efforts to reduce press cycle confirm the important role of initial dewatering. Another possibility especially for lower density panels is convection drying in an oven instead of conductive drying in a hot press or a combination of the two methods. The hybridization of CNF binder with fungal treatment of wood particles promises the possibility of cost reduction attributed to the lower amount of CNF required to achieve acceptable physical and mechanical properties. In our previous studies^25,26^ where CNF was the sole binder to produce particleboard panels, at least 15 wt.% CNF was required to meet ANSI standard minimum levels. Using mycelium treated wood particles the required CNF content was only 2.5% indicating the great potential of hybridizing the two binder systems.

## Conclusions

This study investigated the hybrid systems of wood, mycelium and CNF in the production of fully bio-based composite panels. Two systems of applying fungal biomass were compared and growing mycelia on the wood resulted in better properties than physically mixing pure wood particles and mycelium. Growing mycelium on wood did not change particle dimensions and shape but well covered on the surface of the particles, which had positive effects on bonding. The added CNF formed a uniform film over the particles and improved the physical and mechanical properties of the composites at loadings up to 5% and 2.5%, respectively, for 0.4 g.cm^−3^ and 0.6 g.cm^−3^ composite panels. This system also works in lower density composite systems. Overall, this novel composite system showed good physical and mechanical properties and has potential to replace formaldehyde-based composites. Further improvement of the hybrid system, testing of other properties and other potential mechanisms are the focus of our current studies. Finally the impressive better dimensional stability of composites produced from mycelium-treated wood was promising in terms of potential to produce outdoor-type composites using water-resistant resins.
